# Mesothelioma in Familial Mediterranean Fever With Colchicine Intolerance: A Case Report and Literature Review

**DOI:** 10.3389/fimmu.2020.00889

**Published:** 2020-05-13

**Authors:** Rosa Talerico, Carmine Cardillo, Francesco De Vito, Francesca Schinzari, Manuel Soldato, Maria Cristina Giustiniani, Elena Verrecchia, Raffaele Manna

**Affiliations:** ^1^Department of Internal Medicine and Gastroenterology, Università Cattolica del Sacro Cuore, Fondazione Policlinico Universitario A. Gemelli IRCCS, Rome, Italy; ^2^Department of Pathology, Università Cattolica del Sacro Cuore, Fondazione Policlinico Universitario A. Gemelli IRCCS, Rome, Italy; ^3^Department of Internal Medicine, Rare Diseases and Periodic Fevers Research Centre, Università Cattolica del Sacro Cuore, Fondazione Policlinico Universitario A. Gemelli IRCCS, Rome, Italy

**Keywords:** Familial Mediterranean fever (FMF), malignant mesothelioma (MST), colchicine, peritoneal recurrent inflammation, canakinumab, anakinra, interleukin (IL)-1b inhibition

## Abstract

A 65-year-old Italian physician affected by Familial Mediterranean fever (FMF) was hospitalized due to progressive abdominal enlargement, which had begun 6 months before admission. Physical examination revealed ascites and bilateral leg edema. Abdominal CT scan showed ascitic fluid and extensive multiple peritoneal implants; peritoneal CT-guided biopsy revealed an epithelial-type malignant mesothelioma. The patient's past medical history revealed recurrent episodes of abdominal pain and fever from the age of 2. Clinical diagnosis of FMF was suspected at the age of 25, while genetic analysis, performed at the age of 50, confirmed homozygosity for the M694I mutation in the MEFV gene. Treatment with the first line FMF drug colchicine was started and stopped several times because of worsened leukopenia. The patient in fact had a history of asymptomatic leukopenia/lymphopenia from an early age; the intake of colchicine aggravated his pre-existing problem until the definitive suspension of the drug. As for second-line drugs, canakinumab was first prescribed, but due to prescription issues, it was not possible to be administered. When he was given anakinra, there was a worsening of leukopenia leading to septic fever. Systematic literature review indicates that, in most cases, recurrent peritoneal inflammation results in benign peritoneal fibrosis or less commonly in encapsulating peritonitis. There are only a few reported cases of recurrent peritoneal inflammation progressing from FMF to peritoneal mesothelioma (MST). In such cases, intolerance to colchicine or its erratic intake may lead to long-term recurrent inflammation, which usually precedes the development of the tumor, while pre-existing leukopenia, as in our patient, could also be a factor promoting or accelerating the tumor progression. In conclusion, we suggest that in the presence of intolerance or resistance to colchicine, interleukin (IL)-1 inhibition could suppress peritoneal inflammation and prevent MSTs.

## Introduction

Familial Mediterranean fever (FMF) is an autoinflammatory hereditary disorder characterized by recurrent bouts of fever and serosal inflammation at one or multiple sites (1). It is inherited as a single autosomal recessive trait (2), and common complications include secondary (AA) amyloidosis and peritoneal adhesions.

The goals of FMF treatment are the prevention of acute attacks and the reduction of subclinical inflammation between attacks. The disease and its complications are usually managed with colchicine, which is the standard recommendation in all patients with FMF (3). Approximately 5–10% of FMF patients are colchicine resistant, and 2–5% do not tolerate the drug due to its side effects (4). The currently preferred second-line treatment for these patients is by represented interleukin (IL)-1 inhibitors (canakinumab or anakinra) (5–8).

This case report describes the history of an FMF patient who was intolerant to colchicine, developed MST, and in whom treatment with IL-1 inhibitors was unfortunately delayed.

We hereby discuss the association between Familial Mediterranean fever and the appearance of malignant mesothelioma; very few cases have been described in the literature. Although such association is controversial (9, 10), this and other similar cases lead us to support the relationship of chronic peritoneal inflammation and the development of MST. Colchicine resistance or intolerance promotes chronic inflammation; therefore, IL-1 biological inhibitors, by acting on the inflammatory cascade and its mediators, may prevent amyloidosis as well as recurrent peritoneal inflammation in these patients.

## Case Report

In January 2019, a 65-year-old physician affected by Familial Mediterranean fever was hospitalized at our Center for progressive abdominal enlargement, which had begun 6 months before admission. Physical examination revealed ascites and bilateral leg edema.

Past medical history dates back to the age of 2 with self-limited acute abdominal pain bouts associated with fever. The patient was completely asymptomatic between attacks. These episodes lasted from 24 to 72 h and occurred at unpredictable periods ranging from a weekly interval to twice a year. They were also accompanied by an erysipelas-like erythema and sometimes orchitis.

After becoming a doctor, the patient diagnosed himself with Familial Mediterranean fever (FMF) at the age of 25. In 2003, at the age of 50 the genetic test was performed in our Center and revealed M694I homozygous mutations of the exon 10 of the MEFV gene (mutation c.2082G>A).

The patient had a long history of stable mild leukopenia/lymphopenia since at an early age in the absence of any recurrent infections, without familial history.

Blood exams (white cell count and lymphocyte typing) confirmed this data: WBC 3,680/mm^3^, lymphocytes 1,170/mm^3^. The subtype analysis showed marked reduction in CD3+ T lymphocytes with normal ratio CD4/CD8; CD3+ was 853 mm^3^ (72.4%), CD4+ was 542 mm^3^ (46%), CD8+ was 271 mm^3^ (23 %), CD4/CD8 ratio was 2, CD19+ was 47 mm^3^ (4%), and CD16+ was 2% of the total lymphocytes.

Serology for HIV, EBV, and CMV was negative. Autoantibodies such as ANA, anti-DNA, anti-endomysial, and anti G.P.C. antibodies were absent, and immunoglobulin levels were normal. Mantoux test was repeatedly negative, confirmed by Quantiferon test. Gastroscopy showed chronic gastritis with focal atrophy, *H.P*. negative; Vitamin B12 and folic acid levels were normal.

In addition, as it is usual in FMF patients with a delayed diagnosis, he had had appendectomy at age 20. Interestingly, during a laparoscopic hernioplasty performed at the age of 45, a peritoneal biopsy revealed “*Mononuclear cellular infiltrates with fibrotic appearance*.”

He had always worked in the Emergency Room; no asbestos exposure was evident in his past medical history. After FMF diagnosis was established, multiple trials of 1 mg/day of colchicine treatment were performed, but given the side effect of worsening neutropenia (<1,500 mm^3^) and the consequent risk of infections, it was stopped. As an E.R. physician being exposed to sick contacts, he decided to treat himself with paracetamol or NSAIDs à* la demande* during the attacks.

Since August 2018, the fever had become persistent with monthly recrudescence, and in October 2018, the patient was admitted to another hospital due to atrial fibrillation, ascites, and bilateral leg edema. Peritoneal percutaneous drainage was performed, and fluid cytology analysis revealed “*hyperplastic-dysplastic cellular figures, suggesting uncertain mesothelial proliferation: reactive or neoplastic*.” The patient refused the CT-guided biopsy.

To control the fever, canakinumab, a specific IL-1β (interleukin 1-beta) monoclonal antibody was prescribed in 2018. Owing to regulatory prescription limitations in the region where the patient resided, it was decided that anakinra, an interleukin-1 receptor antagonist (IL-1RA), would be the treatment of choice. When he was given anakinra, there was a worsening of leukopenia leading to septic fever.

**Figure 1:**
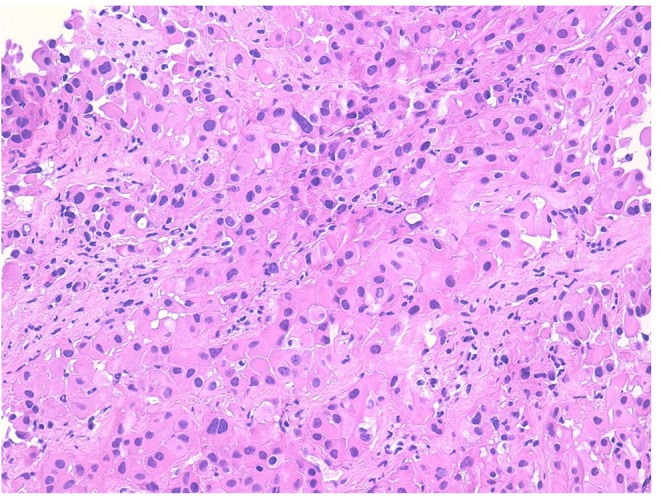
Proliferation of large atypical cells with abundant eosinophilic cytoplasm organized in nests and ribbons (hematoxylin–eosin).

**Figure 2:**
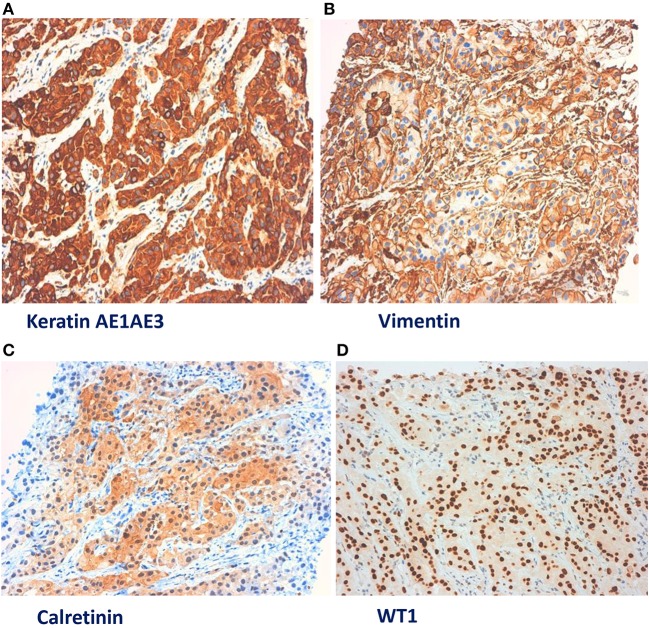
Legend: **(A)** Keratin AE1AE3. **(B)** Vimentin. **(C)** Calretinin. **(D)** WT1.

In January 2019, he was admitted to our hospital (Periodic Fever Research Centre) to investigate the etiology of fever and to exclude a neoplastic origin of ascites. Blood cultures were performed and revealed the presence of *Staphylococcus aureus MRSA*, which was treated with Vancomycin. Abdominal CT scan showed multiple extensive peritoneal lesions, and peritoneal fluid cytology analysis confirmed the presence of neoplastic cells. CT-guided biopsy was performed and a histological diagnosis of epithelioid MST (Figures 1, 2) (phenotype: CK-AE1AE3+, CK5/6+, Calretinin+, Vimentin+, WT1+, BerEp4–) was established. Despite the initial plan of tumor surgical resection plus intraabdominal chemotherapy (HYPEC), the clinical picture deteriorated rapidly with worsening of the general conditions and death after 2 weeks.

## Discussion

Monogenic periodic fever syndromes are rare conditions, which have made possible the discovery of the inflammasome-related pathologies (11) and the effects of recurrent inflammation.

In this setting, FMF is defined as an hereditary autoinflammatory disorder typically encountered in certain Mediterranean populations, such as Jewish, Middle-east, Anatolian Turk and Armenian, Greek, and Italian (12–14).

FMF is characterized by recurrent attacks of fever and serosal inflammation. The mutant FMF gene (MEFV gene) is located on the short arm of chromosome 16 and is known to have pleiotropic effects (15).

The MEFV gene codifies for a protein known as *Pyrin/Marenostrin*, whose mutation has been associated with a gain-of-function (16) that enhances inflammation in response to trigger factors (PAMPs/DAMPs etc.).

An association of MEFV mutations with some neoplasms (as multiple myeloma) has been reported in the literature [it has been speculated that MEFV can act as an onco-suppressor gene (17)], but is not confirmed. FMF usually presents as recurrent or, less commonly, sporadic fever attacks associated with serosal inflammation involving the peritoneum, pleura, or synovia. Sometimes the onset is as an acute abdomen, and recurrent laparotomies are unable to identify the underlying pathology. Acute attacks are typically accompanied by elevation of inflammatory markers such as WBC, CRP, and SAA. FMF should be suspected in all individuals with recurrent febrile episodes associated with peritonitis, synovitis or pleurisy, recurrent erysipelas-like erythema, in an individual with compatible family history or specific ethnic origin (18). Genetic testing is useful to support diagnosis and exclude other autoinflammatory syndromes mimicking FMF. AA amyloidosis (19) is a well-known complication in patients with long-standing FMF before colchicine treatment era. Other long-term complications include peritoneal adhesions due to recurrent peritonitis, leading to small bowel obstruction, infertility, peritoneal fibrosis, encapsulating peritonitis, but rarely, peritoneal MST has been reported (9, 10).

Malignant MST is a rare highly lethal malignancy originating from the lining of pleural, peritoneal, pericardial, or tunica vaginalis serous membranes. One-third of the MSTs are peritoneal in origin, and the most important cause of these tumors is represented by asbestos exposure, whereby incidence rates range from 13 to 100% among exposed population goals (20).

Besides leading to fibrosis and encapsulating peritonitis, the long-standing peritoneal inflammation typical of FMF patients could predispose to MST development as evidenced by this and other case reports described in the literature. Up to this date, several examples of chronic or recurrent inflammatory conditions linked to cancer have been well-established, for instance Barrett's metaplasia with esophageal cancer and HCV infection with hepatocellular carcinoma, among others. Although the exact mechanism by which inflammation leads to cancer development is not well-known, in chronic inflammatory conditions, a combination of DNA repair abnormalities and inappropriate immune response may lead to, and promote, oncogenesis (21).

In this setting, the cell proliferation of repairing tissue damage, with release of substances, promote cell survival, tissue remodeling, and angiogenesis. Mediators produced by activated immune cells may lead to oxidative stress resulting in genomic instability and mutations, promoting further genotoxicity (22). Such mutations arising in chronic inflammation may give origin to malignant proliferations.

In our report, there was neither a history of exposure to asbestos nor first-degree relatives with neoplasms, lymphomas, or MST. Moreover, 20 years before the development of MST in our patient, the histology of peritoneal biopsy documented benign cell infiltrations with fibrotic appearance.

With regard to leuko-lymphopenia, lymphocytic typing showed a marked reduction in CD8+ T– and CD16+ lymphocytes (NK cells). In the absence of ongoing infections, a suggestive medical history of primitive immunodeficiency, leukopenia was considered a benign idiopathic form (23).

Therefore, long-standing recurrent peritoneal inflammation may have played an important role in the pathogenesis of MST, while leukopenia/lymphopenia may have reduced immune surveillance against activated/proliferative mesothelial cells after the onset of DNA mutagenesis.

Since the clinical history of this case raises suspicion of an association between recurrent uncontrolled peritoneal inflammation and increased risk of MST, we searched all FMF cases associated with MST, reported in the literature (Table 1).

**Table 1:** FMF cases associated with MST.

**References**	**Age of patient**	**Type of mesothelioma**	**Asbestos exposure**	**Survival**	**Use of colchicine**
Eryilmaz et al. (24)	43-year-old man	Peritoneal benign cystic mesothelioma (BCM)	No	Survival	Regular
Challita et al. (25)	50-year-old woman	Malignant pleural mesothelioma	No	Not clear	Regular
Sahin et al. (26)	51-year-old man	Malignant peritoneal mesothelioma	No	1 month	Not constant
Hershcovici et al. (27)	Patient 1: 61-year-old man Patient 2: 38-year-old woman	Malignant peritoneal mesothelioma	No	Not mentioned	Patient 1: regular; Patient 2: not constant
Bani-Hani and Gharaibeh (28)	49-year-old man	Malignant peritoneal mesothelioma	No	13 months	Not clear
Curgunlu et al. (29)	25-year-old woman	Peritoneal benign cystic mesothelioma (BCM)	Unknown	Survival	Regular
Belange et al. (30)	60-year-old man	Malignant peritoneal mesothelioma	No	2 months	Not constant
Gentiloni et al. (10)	39-year-old man	Malignant peritoneal mesothelioma	No	6 months	Not constant
Chahinian et al. (31)	Man, age not mentioned	Malignant peritoneal mesothelioma	Not	Not mentioned	Regular

We collected nine cases of peritoneal malignant mesothelioma and two cases of peritoneal benign cystic mesothelioma cases, from which several conclusions can be drawn (10, 24–31):

1. None of the above-mentioned MST patients with FMF had a history of asbestos exposure.
2. Almost all FMF case reports have peritoneal MSTs (except for one case of pleural mesothelioma in a patient with FMF and rheumatoid arthritis), in spite most malignant MSTs affect the pleural membrane in the general population. Such finding can be explained by the fact that in more than 70% of FMF patients, chronic inflammation is localized at the level of the peritoneum, whereas the pleura is involved in only 30% of cases (20, 32, 33), thus reinforcing the hypothesis that local chronic inflammation may have a role in the pathogenesis.
3. Many of these MST cases reported (10, 26, 27, 30) a poor compliance in the intake of colchicine related to its side effects. These cases belong to a period when biological IL-1 inhibitors were not yet available in the drug market.
4. The report of Gentiloni et al. (10) showed a peritoneal MST in one of two FMF brothers; the first one not treated with colchicine developed MST, after 25 years of diagnostic delay. The second one, treated by colchicine without diagnostic delay, never developed MST.
5. Two patients were affected by peritoneal benign cystic mesothelioma (BCM) (24, 29), which is usually considered an entirely different entity with a much better prognosis and onset at a younger age. However, BCM and MST are two conditions histologically different with a distinct course, although having common inflammatory recurrent triggers and perhaps different predisposing factors. Moreover, a long-term follow-up of these BCM patients has not been described.
6. The mean age of onset in malignant MST (50 years) appears to be higher than those of the two patients with BCM (34 years), but this should be related to the duration and severity of FMF and the type of genetic mutation (exon 10 mutations are associated with a more severe disease course).

## Conclusions

We describe a case of peritoneal MST developing in an FMF patient who could not be treated with colchicine. This is the second case of MST in our Center, where 450 FMF patients (mainly from the South and Center of Italy) have been in follow-up since 1997; both patients were not treated with colchicine.

Approximately 5–10% of FMF patients are colchicine non-responders, and 2–5% do not tolerate the drug mainly due to its side effects (4). Consequently, some patients do not take the drug at the prescribed dosage, resulting in poor control of inflammation.

In the clinical practice, the goals of FMF treatment are prevention of acute attacks and suppression of any subclinical inflammation between attacks to reduce the risk of complications. The first-line treatment is represented by colchicine, which is recommended in all FMF patients, regardless of the frequency and intensity of attacks. Intermittent use of high-dose colchicine or only during acute attacks is not recommended since it does not protect from complications associated to low-grade inflammation occurring during asymptomatic intervals (3, 34, 35).

Colchicine affects neutrophilic migration in response to inflammatory stimuli, while blocking mitotic activity occurs at higher dosages than those used in FMF (36); however, no cytostatic effect of colchicine is expected at the common dosage, being a reversible microtubule depolymerizer.

In recent years, the IL-1 inhibitor biologic drugs became available (canakinumab, anakinra, and rilonacept) for treatment of FMF patients with colchicine resistance or intolerance. The choice of IL-1 inhibitor is based on a combination of factors including regulatory or insurance requirements, route of administration, and cost. Canakinumab is generally preferred due to patient's compliance, since it is administered as a subcutaneous injection every 4–8 weeks.

Analysis of the association of FMF and MST described in the literature shows that many of these FMF patients are not compliant with colchicine treatment.

Therefore, in case of colchicine intolerance (as resistance), IL-1 inhibiting biological drugs, acting on the inflammatory cascade and its mediators, could prevent amyloidosis as long-standing recurrent peritoneal inflammation in FMF patients. It is logical to ask what the patient's prognosis would have been if he could have received regular treatment with colchicine or if IL-1 inhibitors had been administered timely many years before the MST diagnosis.

The patient's last wish was for his medical history to be described in detail.

## Data Availability Statement

The datasets generated for this study are available on request to the corresponding author.

## Author Contributions

RT was responsible for drafting the manuscript, reviewing the literature, and discussing the case report. CC, FD, FS, and MS contributed to the management and treatment of the patient. MG analyzed the biopsy findings. EV was responsible of patient's data set. RM was responsible for patient diagnosis and follow-up, discussion and revision of the manuscript.

## Conflict of Interest

The authors declare that the research was conducted in the absence of any commercial or financial relationships that could be construed as a potential conflict of interest.
